# The Psychometric Properties of the Iranian Version of Repetitive Behavior Scale‐Revised (RBS‐R) Questionnaire in Children With Autism Spectrum Disorder

**DOI:** 10.1002/hsr2.70984

**Published:** 2025-07-09

**Authors:** Amir Mohammad Salehi, Shayan Bahadivand Chegini, Fatemeh Esmaeili, Niloofar Rabiei, Ensiyeh Jenabi, Mohammad Rezaei

**Affiliations:** ^1^ Student Research Committee Hamadan University of Medical Sciences, School of Medicine Hamadan Iran; ^2^ Department of Biostatistics, School of Public Health Hamadan University of Medical Sciences Hamadan Iran; ^3^ Mother and Child Care Research Center Hamadan University of Medical Sciences Hamadan Iran; ^4^ Autism Spectrum Disorders Research Center Hamadan University of Medical Sciences Hamadan Iran

**Keywords:** autism, Iran, psychometric, translating, validation studies

## Abstract

**Background and Aims:**

Restricted and repetitive behaviors (RRBs) are fundamental characteristics of autism spectrum disorders (ASD). There is a need for a reliable and valid instrument to evaluate the diversity of RRBs and assess their severity. The Repetitive Behavior Scale‐Revised (RBS‐R) is a recognized tool for screening RRBs. This study aims to investigate the psychometric properties of the RBS‐R questionnaire in Iran by identifying children with ASD who have Persian‐speaking parents.

**Methods:**

In this case–control study conducted in 2024 in Hamadan, 160 children were included: A case group diagnosed with autism (25 girls, 52 boys) and a control group of healthy volunteer children (30 girls, 53 boys) aged 4–12 years. The ASD group exhibited a minimum mean length of utterance (MLU) of 2. Participants diagnosed with intellectual disabilities were excluded from the study. The study assessed the reliability, content validity, and face validity to evaluate the psychometric properties of the tool. Exploratory factor analysis was used to identify questionnaire subscales. All assumptions, including the Kaiser–Meyer–Olkin (KMO) test, Bartlett's test, and question correlations, were checked. Subscales were extracted using ordinary least squares (OLS) and Equamax rotation, with the number of subscales determined based on the Scree plot method and eigenvalues greater than 1.

**Results:**

The study identified six factors (aggressive, focused and self‐harming, resistant to change, obsessive, ritualistic, and insistent), which together explained 51.082% of the total variance. The Cronbach's alpha value for the Persian version of RBS‐R was reported as 0.924. The questionnaire showed a sensitivity of 88.31%, specificity of 83.13%, positive predictive value of 82.93%, negative predictive value of 88.46%, and an area under the curve of 91.7%. The selected cutoff value in this study was determined to be 17.5.

**Conclusion:**

The Persian version of the RBS‐R is valid and reliable. It also demonstrated acceptable internal consistency and sensitivity, suggesting that it can be used as a screening measure for ASD in Iran.

**Abbreviations**
ASD: autism spectrum disorder
AUC: area under the curve
CVI: content validity index
CVR: content validity ratio
DSM‐5: Diagnostic and Statistical Manual of Mental Disorders, 5th edition
EFA: exploratory factor analysis
KMO: Kaiser–Meyer–Olkin
NPV: negative predictive value
OLS: ordinary least squares
PPV: positive predictive value
RBBs: restricted and repetitive behaviors
RBS‐R: Repetitive Behavior Scale‐Revised
ROC: receiver operating characteristic
SD: standard deviation

## 1 Introduction

Autism spectrum disorder (ASD) is a group of neurodevelopmental conditions characterized by impaired social communication and restricted behaviors [1]. These disorders encompass a broad range of restricted and repetitive behaviors (RRBs) and stereotyped behaviors associated with ASD [2]. Genetic factors account for 20%–30% of ASD cases, whereas the remaining 70%–80% are attributed to a complex interaction between environmental risk factors and genetics [3]. ASD can be identified between 18 and 24 months of age, although diagnoses tend to be more reliable after the age of 3 [4, 5]. Numerous studies emphasize the importance of early screening, which enhances child outcomes by prioritizing early identification rather than waiting until the age of 3 [6, 7, 8, 9].

RRBs refer to a range of behaviors (e.g., rubbing, kicking, and rocking) that are characterized by their repetitive nature, which may occur at varying frequencies. Although these behaviors do not directly lead to a strong insistence on sameness, one aspect of restricted behavior involves insisting that things are done in the same way each time [10]. In the Diagnostic and Statistical Manual of Mental Disorders, 5th edition (DSM‐5), RRBs are classified into four subtypes. The first subtype involves the repetition of actions, speech, or the use of objects, such as turning around and parroting. The second subtype emphasizes insisting on sameness, for example, walking the same route each time. The third subtype encompasses restricted interests, such as an obsession with wheels. The final subtype involves sensory processing abnormalities, like smelling people's hair [11].

The importance of differential assessment and diagnostic tools to evaluate the severity of behaviors in ASD has been widely emphasized in numerous studies worldwide. Common methods for measuring RRBs in ASD include observational techniques, caregiver interviews, and standardized questionnaires. A significant challenge in assessing RRBs lies in the wide variability of these behaviors among children with ASD [12]. Various instruments have been developed to measure ASD symptomology, such as the Sameness Questionnaire, the Abnormal Focused Affections Checklist, and the Childhood Routines Inventory [13]. However, these tools were not specifically designed to assess RRBs in ASD and often capture only specific aspects of these behaviors.

In recent years, researchers have made significant strides in understanding patients' conditions and their clinical referrals by creating and enhancing ASD screening tools [10]. One such tool is the Repetitive Behavior Scale‐Revised (RBS‐R) questionnaire [14], which assesses a range of intermittent RRBs considered key symptoms of this disorder. The questionnaire consists of 43 questions, dividing these behaviors into six subgroups. Participants score each question from 0 to 3, resulting in a total score ranging from 0 to 129. Higher scores indicate greater RRBs [14, 15]. The psychometric characteristics of the RBS‐R have been examined across various studies involving participants ranging from 16 months to 63 years old [16]. These studies included individuals diagnosed with ASD [17, 18, 19] and those with intellectual disabilities [20]. Research on the RBS‐R's psychometric properties has been conducted on versions of the scale translated into multiple languages, including English, Spanish, Italian, Japanese, Greek, German, and Malay [13, 14, 16, 18, 19, 20, 21, 22, 23].

Due to the lack of a Persian version of this questionnaire, there has been no research on the use of the RBS‐R and its applicability for accurately identifying individuals with ASD among Persian‐speaking populations. Studies conducted in other countries indicate that the RBS‐R questionnaire has high reliability and stability for identifying children, adolescents, and adults with ASD. Therefore, this study aimed to estimate the psychometric properties of the RBS‐R in identifying Persian‐speaking children with ASD in Hamadan, Iran.

## 2 Materials and Methods

### 2.1 Design of the Study and Participants

In Hamadan City, located in the western part of Iran, a case–control study was conducted between December 2023 and March 2024. The study protocol was reviewed and approved with the code IR.UMSHA.REC.1402.366 by Hamadan University of Medical Sciences. Parents and guardians of the participating children, as well as the staff members, were provided with a detailed explanation of the study's objectives. All information, including participants' names, was guaranteed to remain confidential, and informed consent was obtained.

The study included children aged 4–12 who had been diagnosed with autism based on DSM‐5 criteria. A total of 160 children participated in the study, comprising 77 (48.1%) diagnosed with autism and 83 (51.9%) in the control group. All children were evaluated using the Autism Diagnostic Interview‐Revised (ADI‐R) and assessed by a psychiatrist to confirm the diagnosis. To minimize the impact of cognitive deficits, participants diagnosed with intellectual disabilities were excluded from the study, as determined by the third edition of the Wechsler Intelligence Scale for Children (WISC‐III) [24]. The control group consisted of healthy volunteers who were invited to participate in the study. The two groups were matched based on location and age. When forming the control group, it was crucial to include participants who showed no signs of language deficits and had a verified history free of language‐related issues. The assessment of the participants' language abilities was conducted using the Children's Communication Checklist‐Persian (CCC‐Persian) [25]. Candidates were excluded from the control group if they exhibited difficulties with social skills, emotional regulation, neurological conditions, or sensory impairments, including auditory or visual deficits. Additionally, participants in the sample group needed to demonstrate a minimum mean length of utterance (MLU) of 2, indicating a sentence length of two words or more. Those with comorbid neurological conditions, such as seizures, or any sensory impairments that might affect the study's results, were also excluded from the sample group.

### 2.2 RBS‐R Questionnaire

The RBS‐R questionnaire was developed by Bodfish et al. [26]. The RBS‐R serves as a valuable tool for the detailed evaluation of repetitive behaviors (RRBs) in individuals with ASD. It is the most comprehensive and specific instrument available for quantifying RRBs, as supported by various studies that have utilized the RBS‐R to effectively explore subcategories of these behaviors and assess their presence in clinical trials evaluating ASD treatments. Since the performance of a range of intermittent and restricted behaviors (RRBs) is one of the main symptoms of ASD, this questionnaire was created with 43 items across six subscales: stereotype, self‐injury, compulsive behaviors, ritualistic behaviors, sameness, and limited interests. The items are rated using a Likert scale, where a score of 0 indicates that the “behavior does not occur,” and a score of 3 indicates that the “behavior occurs and is a severe problem.” Elevated scores on the RBS‐R indicate a greater severity of repetitive behaviors. The scale is designed to be completed by individuals who are closely acquainted with the child, such as parents, caregivers, or professionals, who are asked to document the child's behaviors observed over the previous month.

### 2.3 Statistical Plan

To meet the objectives of this research, the data set was explored in multiple stages. In the descriptive statistics section, the details of the participants were discussed. A systematic procedure was then followed for translating the questionnaire with linguistic correctness and cultural adequacy. Experts translated the questionnaire to ensure accuracy and then back‐translated it with another team to detect inconsistencies. Both drafts were reviewed by a group of experts to resolve inconsistencies and enhance cultural sensitivity. Face and content validity were assessed to validate the correctness and utility of the questionnaire. Face validity evaluates whether a questionnaire appears to measure a construct, whereas content validity tests how well a questionnaire captures the intended constructs.

In the subsequent step, exploratory factor analysis (EFA) was applied to identify underlying factors that define relationships between observed variables. EFA groups related variables into factors to reveal the underlying structure of the data. Major steps in this process include testing appropriateness, factor extraction, rotation, and determining the number of factors on statistical grounds, all detailed under structural validity. For each extracted factor, Cronbach's alpha coefficient was computed. Additionally, the receiver operating characteristic (ROC) curve and area under the curve (AUC) values were calculated to examine the ability of the questionnaire to distinguish between groups. Sensitivity, specificity, positive predictive value (PPV), and negative predictive value (NPV) were also calculated, as detailed in the last section. All analyses were performed using SPSS software version 16, with statistical significance set at a *p*‐value < 0.05, using two‐sided testing. Further details of each procedure can be found in their respective sections.

### 2.4 Descriptive Analysis

In this study, sociodemographic variables are described in terms of frequency and percentage, whereas quantitative variables are presented as mean ± standard deviation. The means of the quantitative variables (child's age, MLU, mother's age, and father's age) were compared between the two groups—children with autism and the control group—using the Mann–Whitney *U* test and independent samples *t*‐tests. The chi‐squared test was used to examine the relationship between the genders of children in both groups.

### 2.5 Translation

Permission from the developer was obtained via email before the translation. The English version of the questionnaire was independently translated into Persian by two individuals fluent in both languages. Equivalents for words or terms in the English text were documented for use in subsequent steps. The translators were instructed to avoid literal translations. As a result, two independent Persian translations of the questionnaire were produced. In the next step, after discussing the accuracy of the statements in terms of conceptual clarity, the study team compiled a single Persian version of the questionnaire. This Persian version was then translated back into English by two additional professionals fluent in both languages. Since the original English version was not accessible to them, they worked from the Persian translation. After reviewing and correcting any inaccuracies, a single English version of the survey was created and forwarded to the questionnaire developer for approval.

### 2.6 Face and Content Validity

To ensure qualitative face validity, the translated version of the questionnaire was reviewed by 10 parents of children with autism, who provided feedback. The parents involved in this phase were not the same as those participating in the study. They also discussed the importance of each question in the survey. Importance was rated on a scale of 1 to 5, with 5 indicating “*very important*” and 1 indicating “*not important at all*.” Items with an impact score of 1.5 or higher were retained [27].

To assess content validity, 10 experts were invited to evaluate the questionnaire's content in terms of grammar, word appropriateness, and sentence placement. The research team incorporated changes based on the experts' comments where necessary. Each expert also rated each item using a 4‐point scale, where 1 indicated “*not relevant*” and 4 indicated “*highly relevant*.” The relevance of the 43 items was determined using the content validity index (CVI); items with a CVI value of 0.7 or higher were retained [28]. To measure the content validity ratio (CVR), experts reviewed each item and categorized it as “necessary,” “useful but not necessary,” or “not necessary.” According to Lawshe, items with a CVR value of 0.85 or higher were retained [29].

### 2.7 Structural Validity

Since there was no Persian version or cross‐cultural survey of this questionnaire, we first translated the questionnaire and then evaluated the strength of the Persian version using various indices based on different stages of cross‐cultural evaluations. The number of factors or items in each factor may vary across different language versions of the questionnaire. Consequently, finding the best translated questionnaire with the most similarity to the original version is the goal of EFA in such studies. In guidelines for cross‐cultural survey design, several steps such as translation and back‐translation, expert review, appropriateness checks, item analysis with reliability analysis, and EFA have been introduced in various articles [21, 30, 31, 32].

Before conducting EFA and examining patterns in the data, several assumptions needed to be checked. One key question is whether the sample size is adequate. We used the Kaiser–Meyer–Olkin (KMO) test to determine if the sample size was sufficient. A KMO value between 0.70 and 0.80 is considered moderate, a value between 0.80 and 0.90 is regarded as good, and a value above 0.90 is excellent [33]. The next issue to address is whether the items are correlated. Bartlett's test of sphericity assesses whether the correlation matrix significantly differs from an identity matrix. A *p*‐value lower than 0.05 suggests that the items are correlated rather than uncorrelated [34]. An alternative method to evaluate item correlations is to use an anti‐image matrix, where the values on the diagonal must exceed 0.5, indicating that the off‐diagonal values should be relatively small, reflecting weak correlations [35, 36]. Additionally, it is important to consider the strength of correlations within the data matrix. If the correlation between items is weak (i.e., less than 0.30), factor analysis should not be performed [34]. Once all assumptions were confirmed, the ordinary least squares (OLS) method was used to extract the factors. The number of factors was determined based on the Scree plot method, eigenvalues greater than 1, Velicer's Minimum Average Partial (MAP) test, and parallel analysis [37, 38].

### 2.8 Reliability

Following the identification of the factors, we employed Cronbach's alpha coefficient to assess the reliability of each factor and the total questionnaire. There are varying opinions on the acceptable value for alpha. Some suggest it should be at least 0.70, whereas others advocate for a cutoff as high as 0.95. Nunnally suggested that a reliability coefficient of 0.50 or 0.60 is sufficient for initial research, such as exploratory analysis; however, in later editions, the standard was raised to 0.70 [39].

### 2.9 Sensitivity, Specificity, PPV, and NPV Indices

In a binary classification scenario, there are four possible outcomes: true positive (TP), true negative (TN), false positive (FP), and false negative (FN). Table 1 summarizes these scenarios. Sensitivity, specificity, PPV, and NPV are defined as follows: sensitivity (TP/[TP + FN]), specificity (TN/[TN + FP]), PPV (TP/[TP + FP]), and NPV (TN/[TN + FN]). When the index values are close to 1, it indicates that the questionnaire performed well in classification [40].

**Table 1:** The result of a binary classification scenario.

		Individual predicted status by questionnaire	
		Patient	Healthy control
Individual real status	Patient	TP	FN
	Healthy control	FP	TN

### 2.10 Ethical Considerations

This study commenced after receiving the necessary introduction letters from the relevant authorities and obtaining the Code of Ethics (IR.UMSHA.REC.1402.366). All parents were included in the study only if they provided informed consent. All information was collected while maintaining confidentiality and without identifying the names of the patients. Consent was obtained from all participants.

## 3 Results

### 3.1 Descriptive Results

According to the results presented in Table 2, boys constituted the majority of participants in this study, making up 65.0% of the total. The average ages (with standard deviations) of children in the autism and control groups were 6.95 years (SD = 2.83) and 7.38 years (SD = 2.54), respectively. No significant difference was observed between the average ages of the two groups (*p* = 0.16). Furthermore, the average ages of mothers (*p* = 0.22) and fathers (*p* = 0.98) in the study groups did not differ significantly.

**Table 2:** Demographics characteristics in two groups (autism and control).

Quantitative variables		Group				Total		*p*
		Autism		Control				
		Mean	SD^a^	Mean	SD^a^	Mean	SD^a^	
Child's age		6.95	2.83	7.38	2.54	7.17	2.68	0.16^b^
MLU		2.38	1.81	—	—	2.38	1.81	—
Mother's age		38.64	5.53	39.44	4.61	38.98	5.15	0.22^c^
Father's age		41.74	7.24	41.82	6.86	41.76	7.01	0.98^c^

Sociodemographic variables		Frequency	Percent	Frequency	Percent	Frequency	Percent	*p*
Gender	Female	25	32.50	30	36.10	55	34.40	0.63
	Male	52	67.50	53	63.90	105	65.60	
Total		77	48.1	83	51.9	160	100.0	

^a^ Standard deviation.

b Mann–Whitney test.

c Two independent sample *t*‐test.

### 3.2 Exploratory Analysis

Before conducting the EFA, a power analysis was performed using G*Power software. The analysis set the alpha error probability at 0.05, with six predictors and a sample size of 160. This analysis was conducted with various effect sizes ranging from 0.1 to 0.4. The power values corresponding to these effect sizes were between 0.853 and 0.999, establishing that the sample size for this study was adequate to achieve the research objectives. Questions 20, 34, and 35 were excluded due to their low commonalities.

Before conducting the factor analysis, we assessed the necessary assumptions. The KMO value was 0.747, and Bartlett's test yielded a *p*‐value of less than 0.0001. The diagonal values in the anti‐image correlation matrix were all greater than 0.5, indicating adequate sampling adequacy. The correlations among the items were strong, exceeding 0.3. In this study, six factors were extracted using the OLS method and equamax rotation. The rotated loadings, eigenvalues, and the proportion of variance explained for each factor are presented in Table 3, with the total explained variance for these six factors amounting to 51.082%.

**Table 3:** The eigenvalues, rotated loadings, and the proportion of variance explained by six extracted factors.

Item	Description	Aggressive	Focused and self‐harming	Resistant to change	Obsessive	Ritualistic	Insistent
1	Body movements	0.471					
3	Finger movements	0.468					
6	Sensory	0.644					
8	Hits against surface	0.548					
9	Hits w/object	0.689					
12	Rubs/scratches	0.552					
13	Inserts finger/object	0.518					
14	Picks skin	0.599					
21	Repeating	0.514					
22	Needs to touch/tap	0.670					
4	Locomotion		0.527				
5	Object usage		0.512				
7	Hits w/body		0.486				
10	Bites self		0.815				
11	Pulls hair/skin		0.689				
37	Difficult transitions		0.449				
41	Attached to object		0.489				
42	Preoccupied with part of object		0.508				
43	Preoccupation with movement		0.501				
2	Head movements			0.566			
15	Ordering			0.459			
29	Placement of objects			0.520			
30	No new places			0.488			
31	No interruption			0.506			
40	Preoccupation with subject			0.597			
16	Completeness				0.464		
17	Washing				0.708		
18	Checking				0.536		
25	Self‐care routine				0.635		
27	Play/leisure routine				0.416		
28	Communication				0.429		
19	Counting					0.528	
23	Eating/mealtime					0.311	
24	Sleeping/bedtime					0.754	
26	Transportation routine					0.574	
32	Walks certain way					0.583	
36	Videotapes					0.306	
33	Sits certain place						0.391
38	Insists on routine						0.854
39	Insists on time						0.670
Eigenvalues		11.109	3.586	2.875	1.947	1.872	1.856
% of variance		11.611	11.000	8.808	6.922	6.442	6.299

The mean values and standard deviations for the individual items, along with the correlations of the items with the extracted factors, are listed in Table 4. Additionally, the value of Cronbach's alpha for each factor is displayed in the last column.

**Table 4:** Descriptive statistics of items, correlations, and Cronbach's alpha values of six extracted factors.

Item	Description	Mean	SD^a^	Correlation	Cronbach's alpha
*Aggressive*					
1	Body movements	0.71	0.967	0.661	0.885
3	Finger movements	0.76	1.032	0.694	
6	Sensory	0.57	0.897	0.662	
8	Hits against surface	0.33	0.782	0.465	
9	Hits w/object	0.17	0.507	0.422	
12	Rubs/scratches	0.10	0.318	0.34	
13	Inserts finger/object	0.23	0.556	0.421	
14	Picks skin	0.18	0.463	0.353	
21	Repeating	0.55	0.869	0.546	
22	Needs to touch/tap	0.58	0.852	0.648	
*Focused and self‐harming*					
4	Locomotion	0.63	0.831	0.562	0.884
5	Object usage	0.75	0.930	0.595	
7	Hits w/body	0.37	0.791	0.441	
10	Bites self	0.21	0.578	0.422	
11	Pulls hair/skin	0.25	0.661	0.439	
37	Difficult transitions	0.66	0.899	0.657	
41	Attached to object	0.7355	0.90509	0.557	
42	Preoccupied with part of object	0.6323	0.92596	0.639	
43	Preoccupation with movement	0.7419	1.03094	0.688	
*Resistant to change*					
2	Head movements	0.55	0.884	0.582	0.784
15	Ordering	0.71	0.897	0.445	
29	Placement of objects	0.73	0.840	0.529	
30	No new places	0.61	0.907	0.539	
31	No interruption	1.08	1.016	0.615	
40	Preoccupation with subject	1.03	1.072	0.668	
*Obsessive*					
16	Completeness	0.47	0.800	0.515	0.712
17	Washing	0.34	0.734	0.483	
18	Checking	0.30	0.646	0.349	
25	Self‐care routine	0.55	0.854	0.513	
27	Play/leisure routine	0.85	0.881	0.53	
28	Communication	0.71	0.814	0.602	
*Ritualistic*					
19	Counting	0.26	0.601	0.403	0.700
23	Eating/mealtime	0.61	0.872	0.563	
24	Sleeping/bedtime	0.64	0.911	0.580	
26	Transportation routine	0.45	0.749	0.496	
32	Walks certain way	0.32	0.711	0.40	
36	Videotapes	1.03	0.996	0.545	
*Insistent*					
33	Sits certain place	0.28	0.564	0.610	0.722
38	Insists on routine	0.28	0.544	0.723	
39	Insists on time	0.25	0.554	0.638	
Total					0.924

^a^ Standard deviation.

**Figure 1:**
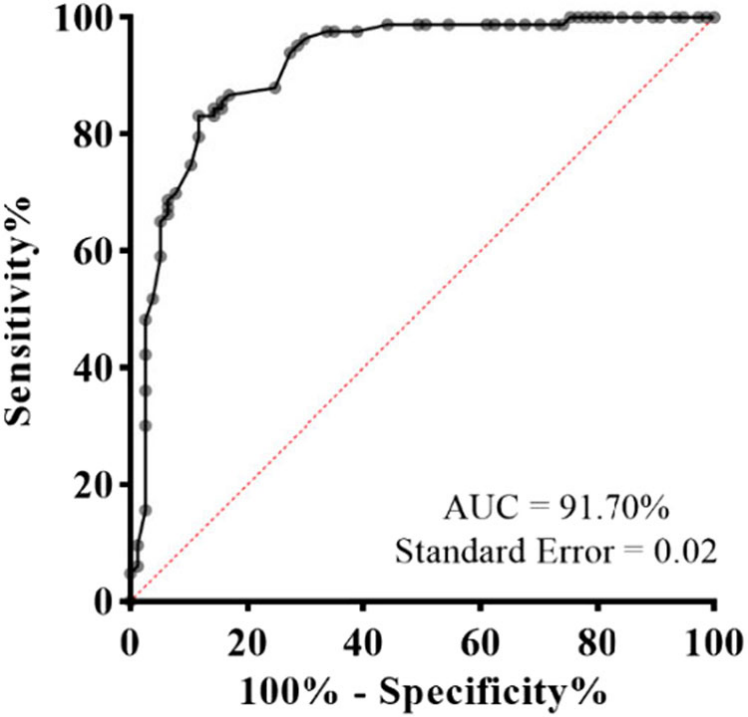
ROC curve and corresponding AUC value.

To evaluate the effectiveness of the questionnaire, ROC curve analysis and Youden's J index were employed to identify the optimal threshold for group separation. The resultant ROC curve is depicted in Figure 1, with a cut‐point value established at 17.5. The misclassification rate was calculated at 14.3%. Sensitivity, specificity, PPV, and NPV are detailed in Table 5.

**Table 5:** Sensitivity, specificity, positive predictive value, and negative predictive value of RBS‐R questionnaire.

Factors	Sensitivity (%)	Specificity (%)	PPV (%)	NPV (%)
Aggressive	84.42	90.36	89.04	86.21
Focused and self‐harming	83.12	95.18	94.12	85.87
Resistant to change	88.31	77.11	78.16	87.67
Obsessive	68.83	66.27	65.43	69.62
Ritualistic	72.73	67.47	67.47	72.73
Insistent	51.00	60.24	55.00	57
Total score	88.31	83.13	82.93	88.46

## 4 Discussion

This study aimed to investigate the psychometric characteristics of the Persian version of the RBS‐R questionnaire. The impetus for this research stems from three key factors: (1) The significant increase in ASD prevalence in recent decades [41], (2) the critical importance of early diagnosis in enhancing therapeutic interventions, particularly for children [42], and (3) the pressing need for culturally appropriate ASD screening tools for Farsi speakers [43]. We translated the RBS‐R into Persian for the first time and utilized it to assess RRBs associated with ASD, conducting exploratory analysis and standardization of the translated version.

Our findings indicate that the Persian version of the RBS‐R is valid, reliable, and demonstrates high internal consistency, with a Cronbach's alpha of 0.924. In comparison, the Chinese version of the RBS‐R exhibits a reliability coefficient of 0.749 and a Cronbach's alpha of 0.834 [10], whereas the Malaysian version reports a validity of 0.97 and internal consistency (Cronbach's alpha) ranging from 0.758 to 0.914 [13].

ASD affects individuals across all social, economic, racial, and ethnic backgrounds. However, disparities in diagnosis rates persist, likely due to limited healthcare access. Additionally, ASD is diagnosed more frequently in males, with a male‐to‐female ratio of nearly 3:1 [44].

According to the Autism and Developmental Disabilities Monitoring (ADDM) Network, the estimated prevalence of ASD in 2020 was 27.6 cases per 1000 children aged 8 years, ranging from 23.1 in Maryland to 44.9 in California. This marks an increase from previous ADDM estimates [45, 46]. Currently, approximately 17% of US children aged 3–17 are diagnosed with neurodevelopmental disorders, including autism, attention deficits, and hyperactivity [47]. Globally, ASD prevalence is estimated at 1 in 36 children, though reported rates are lower in most other regions. The rise in ASD diagnoses is largely attributable to improved screening and diagnostic tools [48].

Recognizing RRBs as a key factor in diagnosing ASD, it is essential to utilize valid and standardized instruments to assess these behaviors. The RBS‐R questionnaire is one such tool, renowned for its accuracy in measuring RRB and providing detailed reports on the presence and intensity of these behaviors [49].

In contrast to the studies conducted by Mirenda et al. [22] and Hooker et al. [49], which employed the original version of the RBS‐R questionnaire, our study necessitated cultural and content localization of the questions. It is crucial to adapt and revalidate any questionnaire following translation for use in a new cultural context [50]. Consequently, our study allocated a portion of its efforts to addressing this issue. During this localization process, although no questions were eliminated based on face and content validity criteria, three questions were identified and removed from the exploratory analysis due to a lack of relevance. Subsequently, the remaining 40 questions were restructured into a new category comprising six subgroups, each with varying numbers and orders of questions. These subgroups are designated as follows: (1) aggressive, (2) focused and self‐harming, (3) resistant to change, (4) obsessive, (5) ritualistic, and (6) Insistent. Notably, each subgroup demonstrated acceptable internal consistency, with a Cronbach's alpha of 0.700 or higher.

The suggested minimum values for sensitivity and specificity of a screening tool for diseases in the community are 0.7 [51]. For the Persian version of the RBS‐R questionnaire, the obtained values are 0.88 for sensitivity and 0.83 for specificity. A sensitivity of 0.88 indicates that the Persian RBS‐R demonstrates a superior ability to accurately screen for RRBs associated with ASD compared to other well‐known tools, such as the M‐CHAT, which has a sensitivity of approximately 0.83 [52] and the Q‐CHAT, which has a sensitivity of about 0.73 [51].

The AUC for the Persian RBS‐R was found to be 0.917. The AUC is a widely used measure of diagnostic accuracy, with a value between 0.9 and 1 indicating an excellent diagnostic test [53]. In our research, we determined that a score of 17.5 is appropriate for categorizing participants as having ASD. Studies by Akcamus et al. [54] and Mirenda et al. [22] suggested that higher scores may indicate increased ASD severity, whereas other studies did not provide specific cutoff points [13, 16, 55].

The advantages of using the RBS‐R include a short completion time, the ability to quantify subtle differences in the degree of impairment, and a weak or nonexistent correlation with IQ [22]. In Iran, there is no national ASD screening and registration system, and families often lack knowledge about this disorder. Consequently, there is limited information regarding the IQ of patients. Therefore, this questionnaire serves as a suitable tool for identifying RRBs commonly associated with ASD in Iran. Although the RBS‐R is a valuable means of screening for RRBs associated with ASD, it is essential to evaluate additional symptoms for an accurate diagnosis of ASD. The RBS‐R is crucial for clinicians in recognizing and understanding the RRBs associated with ASD. With its comprehensive and standardized methodology, the RBS‐R facilitates precise screening and enhances intervention planning by emphasizing the nuances of RRBs across various contexts. Thus, it serves as a vital tool for clinicians seeking to deliver personalized care to individuals with autism.

### 4.1 Strengths and Limitations

Due to the limited availability of ASD screening tools in the Persian language, it is imperative that additional screening instruments be translated for use in Iran. This study aimed to address this issue by evaluating the validity and reliability of the Persian version of the RBS‐R for the first time. It proves beneficial for professionals, including occupational therapists, clinical psychologists, and physicians in Iran, aiming to conduct efficient assessments of RRB in individuals with ASD.

During the study, several limitations were encountered, including a small sample size resulting from constraints in examining patients from other centers. Furthermore, traditional cultural attitudes among parents contributed to their reluctance to report abnormal behaviors in their children. The absence of comprehensive medical records for the patients may have also influenced the study's outcomes.

Another limitation of the current study is the lack of comparison of repetitive behaviors in individuals with ASD against other neurodevelopmental conditions.

It is recommended that researchers and advocates in related fields conduct studies with similar objectives in larger populations or in other Persian‐speaking regions, now that the standardized Persian version of the RBS‐R is available for use. Expanding the availability of screening tools will enhance early detection and intervention for autism in Iran.

## 5 Conclusion

The Persian version of the RBS‐R questionnaire, adapted for individuals with ASD, has successfully demonstrated content validity through a rigorous translation process. It achieved an acceptable CVI, requiring only minor revisions before the development of the pre‐final version. Additionally, the pre‐final version attained a satisfactory face validity index without further modifications, leading to the finalization of the questionnaire. Furthermore, the analysis reveals that this version exhibits acceptable internal consistency, underscoring its potential utility as a screening tool for ASD within the Iranian context.

## Author Contributions

**Amir Mohammad Salehi:** conceptualization, methodology, writing – review and editing, writing – original draft, project administration. **Shayan Bahadivand Chegini:** conceptualization, investigation, writing – original draft. **Fatemeh Esmaeili:** conceptualization, investigation, writing – original draft. **Niloofar Rabiei:** formal analysis, investigation. **Ensiyeh Jenabi:** supervision, methodology, writing – review and editing. **Mohammad Rezaeie:** writing – original draft, writing – review and editing.

## Disclosure

No animals were used in this research. All procedures performed in studies involving human participants were by the ethical standards of institutional and/or research committees and with the 1975 Declaration of Helsinki, as revised in 2013.

## Ethics Statement

This study was approved by the Ethics Committee of Hamadan University of Medical Science (IR.UMSHA.REC.1402.366).

## Consent

The authors have nothing to report.

## Conflicts of Interest

The authors declare no conflicts of interest.

## 1 Transparency Statement

The lead author Mohammad Rezaeie affirms that this manuscript is an honest, accurate, and transparent account of the study being reported; that no important aspects of the study have been omitted; and that any discrepancies from the study as planned (and, if relevant, registered) have been explained.

## Data Availability

Because the consent given by study participants did not include data sharing with third parties, anonymized data can be made available to investigators for analysis on reasonable request to the corresponding author.
